# A Missense Mutation of the Gene Encoding Synaptic Vesicle Glycoprotein 2A (SV2A) Confers Seizure Susceptibility by Disrupting Amygdalar Synaptic GABA Release

**DOI:** 10.3389/fphar.2016.00210

**Published:** 2016-07-14

**Authors:** Kentaro Tokudome, Takahiro Okumura, Ryo Terada, Saki Shimizu, Naofumi Kunisawa, Tomoji Mashimo, Tadao Serikawa, Masashi Sasa, Yukihiro Ohno

**Affiliations:** ^1^Laboratory of Pharmacology, Osaka University of Pharmaceutical SciencesOsaka, Japan; ^2^Institute of Laboratory Animals, Graduate School of Medicine, Kyoto UniversityKyoto, Japan; ^3^Institute of Experimental Animal Sciences, Graduate School of Medicine, Osaka UniversityOsaka, Japan; ^4^Nagisa ClinicOsaka, Japan

**Keywords:** synaptic vesicle glycoprotein 2A (SV2A), seizure susceptibility, GABA release, glutamate release, amygdala, pentylentetrazole

## Abstract

Synaptic vesicle glycoprotein 2A (SV2A) is specifically expressed in the membranes of synaptic vesicles and modulates action potential-dependent neurotransmitter release. To explore the role of SV2A in the pathogenesis of epileptic disorders, we recently generated a novel rat model (*Sv2a^L*174*Q^* rat) carrying a missense mutation of the *Sv2a* gene and showed that the *Sv2a^L*174*Q^* rats were hypersensitive to kindling development (Tokudome et al., 2016). Here, we further conducted behavioral and neurochemical studies to clarify the pathophysiological mechanisms underlying the seizure vulnerability in *Sv2a^L*174*Q^* rats. *Sv2a^L*174*Q^* rats were highly susceptible to pentylenetetrazole (PTZ)-induced seizures, yielding a significantly higher seizure scores and seizure incidence than the control animals. Brain mapping analysis of Fos expression, a biological marker of neural excitation, revealed that the seizure threshold level of PTZ region-specifically elevated Fos expression in the amygdala in *Sv2a^L*174*Q^* rats. *In vivo* microdialysis study showed that the *Sv2a^L*174*Q^* mutation preferentially reduced high K^+^ (depolarization)-evoked GABA release, but not glutamate release, in the amygdala. In addition, specific control of GABA release by SV2A was supported by its predominant expression in GABAergic neurons, which were co-stained with antibodies against SV2A and glutamate decarboxylase 1. The present results suggest that dysfunction of SV2A by the missense mutation elevates seizure susceptibility in rats by preferentially disrupting synaptic GABA release in the amygdala, illustrating the crucial role of amygdalar SV2A-GABAergic system in epileptogenesis.

## Introduction

Synaptic vesicle glycoprotein 2A (SV2A) is highly expressed in the brain including the cerebral cortex, limbic regions, and cerebellum, where it modulates action potential-dependent neurotransmitter release (Crowder et al., 1999; Janz et al., 1999; Xu and Bajjalieh, 2001; Custer et al., 2006; Chang and Südhof, 2009). Although the functional mechanisms of SV2A remain to be clarified, it is suggested that SV2A primes synaptic vesicles to fully respond to Ca^2+^ probably by interacting with the Ca^2+^ sensor protein synaptotagmin (Xu and Bajjalieh, 2001; Chang and Südhof, 2009; Nowack et al., 2010). In addition, previous studies suggest that SV2A plays an important role in the pathogenesis and treatment of epileptic disorders. This is because (1) SV2A-knockout mice exhibited severe seizures (Crowder et al., 1999; Janz et al., 1999), (2) SV2A serves as a specific binding site for certain antiepileptics (e.g., levetiracetam and its analogs; Lynch et al., 2004; Pollard, 2008; Kaminski et al., 2009; Correa-Basurto et al., 2015; Klitgaard et al., 2016) and (3) the expressional levels of SV2A are reported to be altered in various epileptic conditions both in animals (e.g., chemically- and electrically induced kindling) and humans (e.g., intractable temporal lobe epilepsy and focal cortical dysplasia; Matveeva et al., 2007; Feng et al., 2009; Ohno et al., 2009a, 2012b; Toering et al., 2009; van Vliet et al., 2009; Crèvecoeur et al., 2014; Serajee and Huq, 2015). Furthermore, a recent clinical study reported that a missense mutation (R383Q) in exon 5 of the *SV2A* gene resulted in intractable epilepsy, involuntary movements, microcephaly and developmental retardation (Serajee and Huq, 2015).

In order to explore the role of SV2A in modulating development of epileptic disorders (epileptogenesis), we recently generated a novel rat model (*Sv2a^L*174*Q^* rat) carrying a missense mutation (L174Q) in the *Sv2a* gene (Tokudome et al., 2016), using gene-driven ENU mutagenesis/MuT-POWER techniques (Mashimo et al., 2008). *Sv2a^L*174*Q^* rats were susceptible to PTZ seizures and to kindling development associated with repeated PTZ treatments or focal electrical stimulation of the amygdala. In addition, the *Sv2a^L*174*Q^* mutation significantly reduced depolarization-induced GABA release in the hippocampus. These findings suggest that SV2A plays a crucial role in the kindling epileptogenesis possibly by interacting GABAergic neurons. However, the detailed mechanisms underlying the regulation of seizure susceptibility by SV2A remain to be clarified.

In the present study, therefore, we further conducted behavioral and neurochemical studies to clarify the mechanisms (e.g., responsible brain regions and influences on synaptic amino acid release) underlying the seizure vulnerability in *Sv2a^L*174*Q^* rats. The present results show that the *Sv2a^L*174*Q^* mutation elevates excitability of the corticolimbic neural circuit, especially in the amygdala, by preferentially disrupting synaptic GABA release, illustrating the crucial role of amygdalar SV2A-GABAergic system in epileptogenesis.

## Materials and Methods

### Animals

Male *Sv2a^L*174*Q^* rats (Tokudome et al., 2016) were obtained from the National BioResource Project-Rat (F344-*Sv2a^m*1*Kyo^* NBRP-Rat No:0668). The *Sv2a^L*174*Q^* rat, carrying a single nucleotide mutation T521A, was first identified in a gene-driven ENU mutagenesis project in Kyoto University (Mashimo et al., 2008). Thereafter, *Sv2a^L*174*Q^* rats were backcrossed more than five generations on the F344/NSlc inbred background to eliminate mutations potentially induced by ENU mutagenesis elsewhere in the genome. Age-matched male F344 rats (Japan SLC, Shizuoka, Japan) were used as the control animal. The animals were kept in air-conditioned rooms under a 12-h light/dark cycle and allowed *ad libitum* access to food and water. All animal experiments were approved by the Animal Research Committees of Osaka University of Pharmaceutical Sciences and were conducted according to the Institutional Committees’ regulations on animal experimentation.

### Evaluation of Seizure Susceptibility

To evaluate the seizure sensitivity, *Sv2a^L*174*Q^* or F344 rats were treated with an intraperitoneal dose of PTZ (30, 35, and 40 mg/kg for *Sv2a^L*174*Q^* rats; 35, 40, 45, and 50 mg/kg for F344 rats). PTZ-induced seizures were evaluated over 20 min after the drug treatment using a 6-point ranked scale as follows, 0: none response, 1: facial automatisms and twitching of the ears and whiskers, 2: convulsive waves propagating axially along the trunk, 3: myoclonic convulsions with a delay, 4: clonic convulsions, 5: repeated powerful clonic-tonic or lethal convulsions (Racine, 1972; Franke and Kittner, 2001; Kudryashov et al., 2007). The incidence of seizures was judged as positive when the animal showed a seizure score of 3 or more.

### Analysis of Fos Protein Expression

To explore causal brain regions for PTZ seizures in *Sv2a^L*174*Q^* rats, expression of Fos protein, a biological marker of neural excitation, by the seizure threshold level of PTZ was analyzed. For this purpose, *Sv2a^L*174*Q^* and F344 rats were cumulatively injected with an increasing dose of PTZ (first dose: 10 mg/kg, second dose: 20 mg/kg, third dose: 30 mg/kg) with 30-min intervals. The incidence of seizures was monitored for 10 min immediately after each PTZ injection using a seizure scale described previously. Since the PTZ-induced seizures were observed only at 30 mg/kg, brain samples were obtained 2 h after the PTZ (30 mg/kg) injection under pentobarbital (80 mg/kg, i.p.) anesthesia.

After fixation with 4% formaldehyde solution, coronal sections (30 μm thickness) were cut from each brain using a Microslicer (DSK-3000, Dosaka, Kyoto Japan). The immunostaining of Fos protein was performed using a previously published method (Ohno et al., 2011, 2012a). Briefly, formalin-fixed sections were immunohistochemically stained with a goat c-Fos antiserum (Santa Cruz Biotechnology Inc., Santa Cruz, CA, USA) by the ABC method. Fos-immunoreactivity was visualized by the diaminobenzidine–nickel staining method and quantified by counting the number of Fos-positive neurons. Brain regions analyzed includes (1) cerebral cortices: mPFC, CgC, MC, SC, AIC, Pir, AuC, PRh-Ent; (2) basal ganglia and limbic regions: AcC, AcS, dlST, dmST, GP, LS, CA, DG, PMCo, BMA, BLA; (3) diencephalon: LHb, PT, AM, CM, VM, AH, PH, DMH (see Paxinos and Watson, 2007).

### *In Vivo* Microdialysis Study

*Sv2a^L*174*Q^* or F344 rats were anesthetized with pentobarbital (40 mg/kg, i.p.) and fixed in a stereotaxic instrument (Narishige, SR-6, Tokyo, Japan). A guide cannula (1 mm diameter) was inserted into a position 2 mm above the amygdala (P: 2.8 mm, L: 4.8 mm, H: -6.1 mm; Paxinos and Watson, 2007) and fixed to the skull using dental cement. After a recovery period of about 1 week, animals with a chronically implanted guide cannula were subjected to microdialysis experiments. Briefly, a dialysis probe (Eicom, A-I-10-02, Kyoto, Japan) was inserted into the amygdala through a guide cannula and aCSF containing (in mM): NaCl 140, KCl 2.4, MgCl_2_ 1.0, CaCl_2_ 1.2, NaHCO_3_ 5.0, was perfused at a flow rate of 1 μL/min using a microperfusion pump (Eicom, ESP-32, Kyoto, Japan). The dialysate samples were collected into a microtube every 10 min (10 μL/sample). To evaluate the depolarization-evoked synaptic release, high concentration (100 mM) K^+^-containing aCSF was perfused for 60 min through the dialysis probe.

The dialysate samples were analyzed for GABA and glutamate levels using a HPLC-ECD system. GABA and glutamate were derivatized with *o*-phthalaldehyde before the HPLC injection and separated on a cation exchange column (Eicom, 3.0φ × 150 mm; Eicompak SC-5ODS, Kyoto, Japan). The mobile phase consisted of 0.1 M phosphate buffer, 5 mg/L EDTA 2Na, pH6.0, with 27% methanol pumped at a flow rate of 500 μL/min. All data were analyzed by using eDAQ Power Chrom (eDAQ Pty Ltd, Denistone East, NSW, Australia). Extracellular GABA and glutamate levels were expressed as a percentage of the basal control level, which was the mean of the three points before the high K^+^ application, in each animal. The AUC of the high K^+^-evoked GABA or glutamate release was also estimated by the trapezoidal approximation method.

### Immunofluorescence Double Staining

*Sv2a^L*174*Q^* or F344 rats were decapitated under pentobarbital (80 mg/kg, i.p.) anesthesia and brains were removed from the skull. After fixation with 4% paraformaldehyde solution for 24 h, the brain was dehydrated and embedded in paraffin. Formalin-fixed and paraffin-embedded amygdaloid tissues were cut into 4 μm thick sections and the sections were subjected to immunofluorescence double staining with anti-SV2A and anti-Gad1 (Ohno et al., 2012b). Sections were incubated with a goat anti-rat SV2A (dilution 1:500, Santa Cruz Biotechnology, Dallas, TX, USA) and mouse anti-human Gad1 (dilution 1:1000, Santa Cruz Biotechnology, Dallas, TX, USA) for 42 h at 4°C, and then with an FITC (green fluorescence)-conjugated rabbit anti-goat IgG secondary antibody (dilution 1:500, Sigma–Aldrich, St. Louis, MO, USA) and TRITC (red fluorescence)-conjugated rabbit anti-mouse IgG secondary antibody (dilution 1:500, Sigma–Aldrich, St. Louis, MO, USA) to probe SV2A and Gad1, respectively. Immunofluorescence images were obtained with a confocal laser scanning microscope (Carl Zeiss Japan, LSM 700 ZEN, Tokyo, Japan). To quantify SV2A and Gad1 expression, digital images of the amygdala were stored and the integrated optical density was measured by computer analysis with ImageJ software (ver. 1.42, NIH).

### Statistical Analysis

Statistical significance of differences between two groups was performed by Mann–Whitney’s *U*-test (behavioral scores) or Student’s *t*-test (Fos and SV2A expression). Comparisons of seizure incidence rate were done by *X*^2^ test. Differences in GABA and glutamate release (*in vivo* microdialysis) were analyzed by two-way ANOVA followed by Tukey’s *post hoc* test. A *P-*value of less than 0.05 was considered statistically significant.

## Results

### Seizure Susceptibility of *Sv2a^L*174*Q^* Rats

**FIGURE 1:**
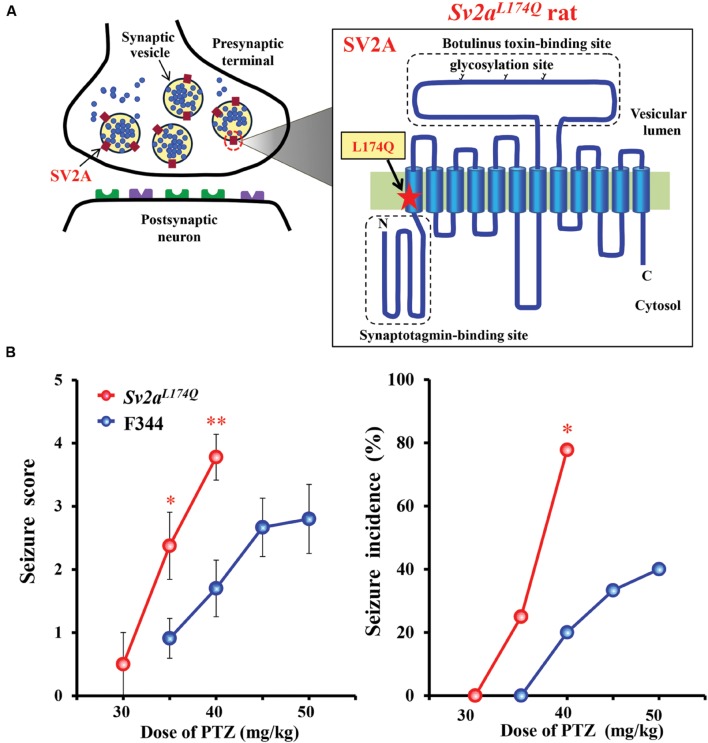
**Seizure susceptibility of *Sv2a^L*174*Q^* rats.** **(A)** Structure of SV2A showing the mutation site L174Q. SV2A possesses a 12-transmembrane structure which contains a putative binding site for synaptotagmin in the N terminal region and glycosylation sites in a long intravesicular loop between transmembrane regions 7 and 8. **(B)** Susceptibility of *Sv2a^L*174*Q^* rats to PTZ-induced seizures. Each point represents the mean ± SEM of 8–11 animals. ^∗^*P* < 0.05, ^∗∗^*P* < 0.01 significantly different from F344 rats.

*Sv2a^L*174*Q^* rats have a missense mutation T521A in the *Sv2a* gene, which results in the substitution of Leu 174 to Gln (**Figure 1A**). The mutation site is located in the first transmembrane region of SV2A that was reportedly essential for the normal function and structure of SV2A (Chang and Südhof, 2009). In order to confirm seizure susceptibility of *Sv2a^L*174*Q^* rats, we evaluated the responses of *Sv2a^L*174*Q^* and F344 rats to PTZ injections (30–50 mg/kg, i.p.). While gross behaviors of *Sv2a^L*174*Q^* rats were normal, these animals showed high susceptibility to PTZ seizures, yielding significantly higher seizure scores at 35 mg/kg [*U*(17) = 20.5, *P* < 0.05] and 40 mg/kg [*U*(17) = 12.0, *P* < 0.01], and a higher seizure incidence at 40 mg/kg (*X*^2^ = 6.34, *P* < 0.05) than F344 rats (**Figure 1B**).

### Fos Expression Analysis

To explore the brain sites most sensitive to the seizure threshold level of PTZ, *Sv2a^L*174*Q^* and F344 rats were cumulatively injected with an increasing dose of PTZ (10, 20, or 30 mg/kg, i.p.) with 30-min intervals. Under these conditions, the first two does did not evoke any seizure either in *Sv2a^L*174*Q^* or F344 rats. However, subsequent 30 mg/kg PTZ induced clonic or tonic-clonic seizures in five out of seven *Sv2a^L*174*Q^* rats while none of seven F344 rats tested exhibited seizures.

**FIGURE 2:**
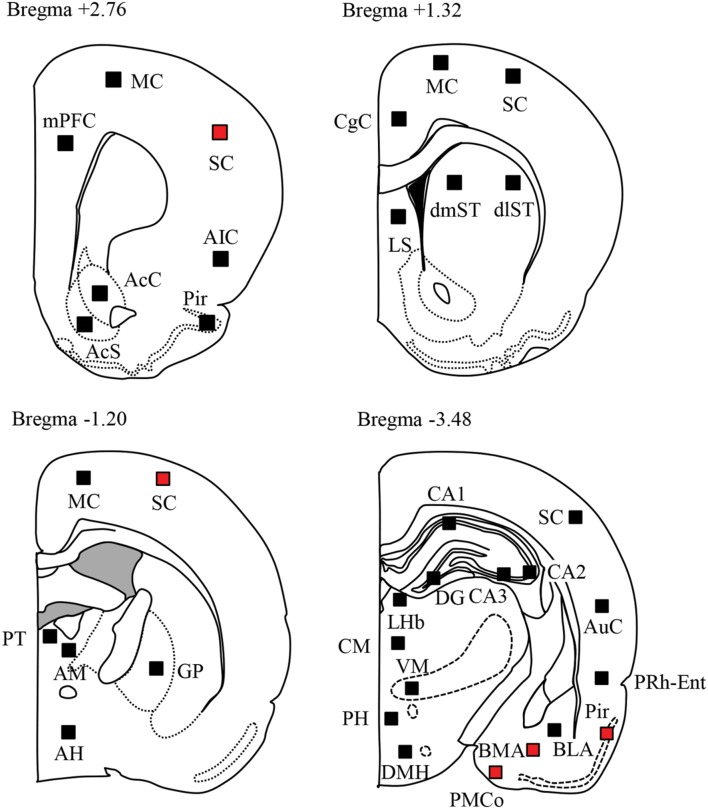
**Topographical Fos expression analysis following PTZ-induced seizures.** Schematic illustrations of the brain sections selected for quantitative analysis of Fos protein expression. Filled boxes in each section indicate the sample areas analyzed. Red boxes represent the regions where Fos expression was significantly elevated by PTZ treatments.

**FIGURE 3:**
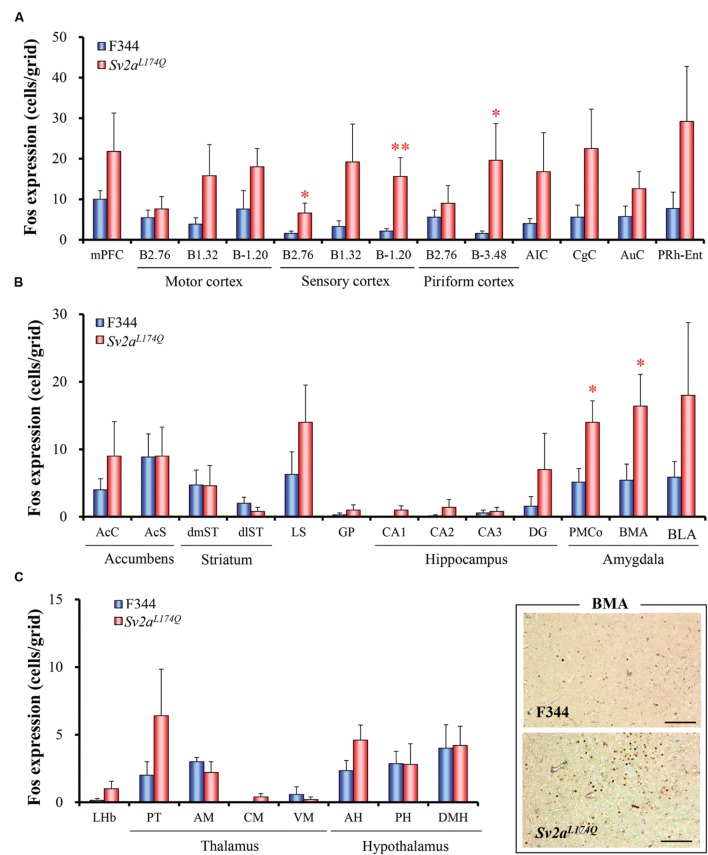
**Regional changes in Fos expression levels by the seizure threshold level of PTZ.** Graphs show Fos expression in the cerebral cortical regions **(A)**, basal ganglia and limbic regions **(B)**, and diencephalon **(C)**. Typical photos of Fos expression in the amygdala are also shown in the right bottom. Each column represents the mean ± SEM of five *Sv2a^L*174*Q^* (PTZ seizure-positive) and seven F344 (PTZ seizure-negative) rats. ^∗^*P* < 0.05, ^∗∗^*P* < 0.01 significantly different from F344 rats. Scale bar: 100 μm.

We next compared the expression of Fos protein, a biological marker of neural excitation, in various regions of the brain between the *Sv2a^L*174*Q^* (PTZ seizure-positive, *N* = 5) and F344 (PTZ-negative, *N* = 7) rats (**Figure 2**). *Sv2a^L*174*Q^* rats showed considerably higher Fos expression than F344 rats in most of the cerebral cortex, reflecting the generalized seizure property of PTZ seizures (**Figure 3A**). PTZ-induced Fos expression in *Sv2a^L*174*Q^* rats were statistically significant in the SC and Pir. On the other hand, the seizure threshold level of PTZ region-specifically elevated Fos expression in the amygdala among 21 subcortical regions examined including basal ganglia, limbic regions, and diencephalon (**Figures 3B,C**).

### GABA and Glutamate Release in the Amygdala

**FIGURE 4:**
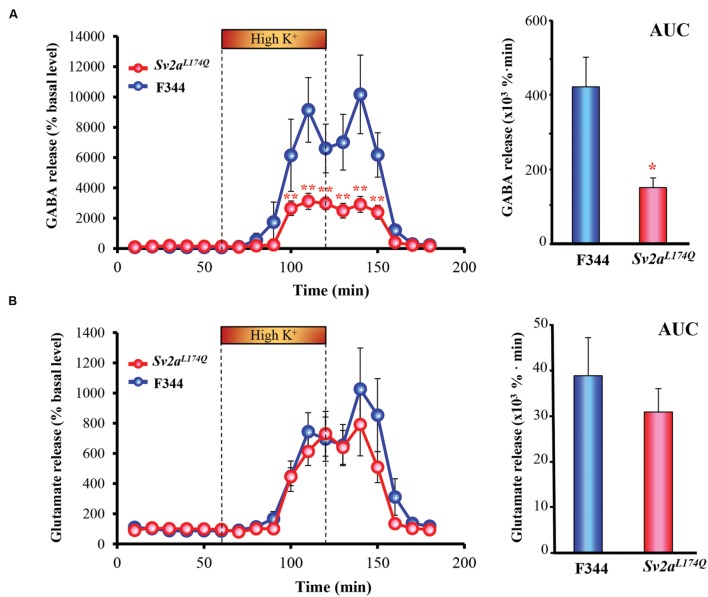
**Synaptic GABA and glutamate release in the amygdala.** **(A)** GABA release. **(B)** Glutamate release. Each point represents the mean ± SEM of 10 or 5 animals. Depolarization stimulation was given by applying high concentration (100 mM) K^+^-containing aCSF (High K^+^) for 60 min through the dialysis probe. AUC (the area under the curve) comparisons of GABA and glutamate release are shown in the right. ^∗^*P* < 0.05, ^∗∗^*P* < 0.01 significantly different from F344 rats. Each point represents the mean ± SEM of 10 or 5 animals.

Since *Sv2a^L*174*Q^* rats exhibited a region-specific excitation of the amygdala by PTZ, we conducted *in vivo* microdialysis studies to evaluate synaptic release of GABA and glutamate in the amygdala. As shown in **Figure 4A**, high K^+^ (depolarization) stimuli evoked GABA release both in *Sv2a^L*174*Q^* and F344 rats. However, the depolarization-evoked GABA release was largely diminished by the *Sv2a^L*174*Q^* mutation [*F*(1,218) = 56.72, *P* < 0.001] (**Figure 4A**). A comparison of AUC also revealed a significant reduction in depolarization-evoked GABA release in *Sv2a^L*174*Q^* rats. On the other hand, in contrast to GABA release, high K^+^-evoked glutamate release was not significantly affected by the *Sv2a^L*174*Q^* mutation (**Figure 4B**).

### SV2A and Gad1 Double Staining in the Amygdala

**FIGURE 5:**
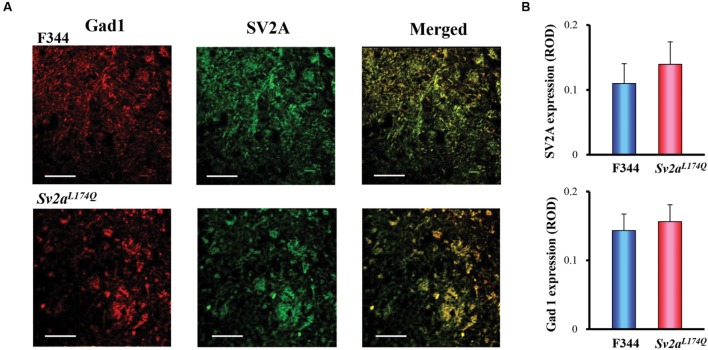
**SV2A and Gad1 double staining in the amygdala.** **(A)** Photos showing representative double staining of Gad1 (left: red), SV2A (center: green) in the amygdala of *Sv2a^L*174*Q^* and F344 rats. Merged photos (right) revealed a predominant co-expression of SV2A with Gad1 (GABAergic neurons) in the amygdala. **(B)** Expression levels of SV2A and Gad1 in *Sv2a^L*174*Q^* and F344 rats. ROD, relative optical density. Each column represents the mean ± SEM of four animals. Scale bar: 50 μm.

We further conducted immunofluorescence double staining of SV2A with Gad1 (also known as GAD67), a marker protein of GABAergic neurons, in the amygdala. As shown in **Figure 5A**, SV2A (green) was mostly co-stained with Gad1 (red) both in *Sv2a^L*174*Q^* and F344 rats, illustrating a specific expression of SV2A in the amygdalar GABAergic neurons. In addition, there were no significant differences in expressional levels of SV2A and Gad1 between *Sv2a^L*174*Q^* and F344 rats (**Figure 5B**).

## Discussion

The present study confirmed that *Sv2a^L*174*Q^* rats carrying a missense mutation, L174Q, in the *Sv2a* gene were highly sensitive to PTZ-induced seizures, supporting the notion that SV2A plays the crucial role in controlling seizure susceptibility. Although a complete deletion of SV2A is known to cause premature death with severe seizure incidence (Crowder et al., 1999; Janz et al., 1999), the behavioral phenotype of *Sv2a^L*174*Q^* rats mimicked those reported in heterozygous SV2A-deficient mice (Kaminski et al., 2009), implying that the *Sv2a^L*174*Q^* mutation causes a partial loss of the SV2A function.

Fos protein is the immediate early gene product and is widely used as a cellular marker of neural excitation. Specifically, brain mapping analysis of Fos expression is useful to identify brain regions related to disease conditions (e.g., epilepsy, emotional disorders and cognitive impairments) or responses to various pathophysiological and pharmacological stimuli (e.g., pain, body temperature, stress, and drug treatments; Morgan and Curran, 1989; Herrera and Robertson, 1996; Ohno et al., 2009b, 2011, 2012a, 2015; Mukai et al., 2013; Fumoto et al., 2014; Iha et al., 2016). In the present study, we treated animals with the seizure threshold dose (30 mg/kg, i.p.) of PTZ which first evoked seizures only in *Sv2a^L*174*Q^* rats. Under these conditions, the PTZ treatment region-specifically elevated Fos expression in the cerebral cortex (e.g., SM and Pir), amygdala. Since PTZ evoked generalized clonic or tonic-clonic seizures associated with a wide-spread excitation of the cerebral cortex (**Figure 3A**), we could not specifically identify the causative regions in the cortical regions. Nonetheless, the present results clearly illustrates that the amygdala is most sensitive to and potential seizure initiation site for PTZ seizures in *Sv2a^L*174*Q^* rats. In addition, *in vivo* microdialysis demonstrated that the *Sv2a^L*174*Q^* mutation preferentially impaired depolarization-evoked synaptic release of GABA in the amygdala without affecting glutamate release. These findings provide important information for our understanding of the SV2A function in modulating seizure susceptibility. Although the hippocampus was less sensitive to PTZ seizures than the amygdala, our results do not deny the potential role of hippocampus in seizure vulnerability of *Sv2a^L*174*Q^* rats since it is known that PTZ evokes seizures by activating limbic regions including the hippocampus (Humpel et al., 1993; Löscher and Ebert, 1996; Szyndler et al., 2009) and that, indeed, a sufficient dose (70 mg/kg, i.p.) of PTZ increases Fos expression in the hippocampus (Bastlund et al., 2005). In addition, we previously demonstrated that the *Sv2a^L*174*Q^* mutation also disrupted depolarization-evoked synaptic release of GABA in the hippocampus. Furthermore, the piriform cortex also seems to be partly involved in the seizure vulnerability in *Sv2a^L*174*Q^* rats since the threshold level of PTZ significantly elevated Fos expression in the posterior part of this structure.

Preferential modulation of synaptic GABA release by SV2A was further supported by the SV2A expression pattern in amygdala GABAergic neurons. In that, most of SV2A-immunoreactivity was expressed in the amygdala neurons and dendrites, which were co-stained with antibodies against SV2A and Gad1. These findings are consistent with our previous findings that SV2A was predominantly expressed in GABAergic neurons, but only rarely in glutamatergic neurons in the mouse hippocampus (Ohno et al., 2012b). Therefore, SV2A seems to specifically regulate synaptic GABA release both in the amygdala and hippocampus. Since the amygdala and hippocampus are potential causative sites for various epileptic disorders (Löscher and Ebert, 1996; Morimoto et al., 2004; Avoli and de Curtis, 2011), the SV2A-GABAergic system in these structures is likely to be involved in pathogenesis of SV2A-related epileptic disorders.

The detailed mechanisms underlying SV2A dysfunction by the *Sv2a^L*174*Q^* mutation are currently unknown. Interestingly, a previous study showed that the neighboring missense mutations (D179A and E182A) of the two charged polar amino acids to the non-polar alanine abolished the normal function of SV2A, possibly by disrupting the protein folding and/or trafficking into synaptic membranes (Chang and Südhof, 2009). Thus, the changes in polarization of the first transmembrane region by the substitution of hydrophobic non-polar Leu 174 to polar Gln may impair the integrity of SV2A function. In addition, we previously showed that the *Sv2a^L*174*Q^* mutation specifically reduced the expression level Syt1, the Ca^2+^ sensor protein modulating synaptic release, among exocytosis regulatory proteins examined (Tokudome et al., 2016). Since SV2A is suggested to prime synaptic vesicles by interacting with the Syt1 (Xu and Bajjalieh, 2001; Chang and Südhof, 2009; Nowack et al., 2010), the disruption of synaptic GABA release by the *Sv2a^L*174*Q^* mutation may result from reduced expression of Syt1. Further studies are required to delineate molecular mechanisms of SV2A dysfunction by the *Sv2a^L*174*Q^* mutation.

The present study supports the clinical view that dysfunction of SV2A is involved in the pathogenesis of epilepsy, including intractable temporal lobe epilepsy and focal cortical dysplasia epilepsy (Feng et al., 2009; Toering et al., 2009; van Vliet et al., 2009; Crèvecoeur et al., 2014). Indeed, a recent study showed that a missense mutation R383Q in the *SV2A* gene caused intractable epilepsy and involuntary movements, which were accompanied by developmental retardation (Serajee and Huq, 2015). Thus, the *Sv2a^L*174*Q^* rat may be useful for exploring the epileptogenic mechanisms of SV2A-related epileptic disorders. Furthermore, since SV2A is known as a specific binding site for certain antiepileptics (e.g., levetiracetam, brivaracetam, and seletracetam; Lynch et al., 2004; Pollard, 2008; Kaminski et al., 2009; Correa-Basurto et al., 2015; Klitgaard et al., 2016), the *Sv2a^L*174*Q^* rat may also be useful as a novel animal model for analyzing the action mechanisms of the levetiracetam-analogs.

## Conclusion

We confirmed high susceptibility of *Sv2a^L*174*Q^* rats to PTZ seizures. Treatment of *Sv2a^L*174*Q^* rats with PTZ at threshold level specifically elevated Fos expression in the amygdala, suggesting that the amygdala is the potential site responsible for seizure vulnerability in *Sv2a^L*174*Q^* rats. In addition, the *Sv2a^L*174*Q^* mutation preferentially reduced depolarization-evoked GABA, but not glutamate, release in the amygdala. The preferential disruption of GABA release due to the *Sv2a^L*174*Q^* mutation was supported by the specific expression of SV2A in GABAergic neurons. The present study suggests that dysfunction of SV2A by the missense mutation elevates seizure susceptibility by disrupting amygdalar synaptic GABA release, illustrating the crucial role of the SV2A-GABAergic system in epileptogenesis.

## Author Contributions

YO designed research. KT, TO, SS, RT, NK, TM, and YO performed pharmacological and neurochemical research. KT, TO, SS, RT, NK, TM, and YO analyzed data. KT, TM, TS, MS, and YO wrote the paper.

## Conflict of Interest Statement

The authors declare that the research was conducted in the absence of any commercial or financial relationships that could be construed as a potential conflict of interest.

The reviewer TK and handling Editor declared their shared affiliation, and the handling Editor states that the process nevertheless met the standards of a fair and objective review.

## Abbreviations

AcC: core region of nucleus accumbens
AcS: shell region of nucleus accumbens
aCSF: artificial cerebrospinal fluid
AIC: agranular insular cortex
AH: anterior hypothalamus
AM: anteromedial thalamic nucleus
AUC: area under the curve
AuC: auditory cortex
BLA: anterior basolateral amygdaloid nucleus
BMA: anterior basomedial amygdaloid nucleus
CA: Cornu Ammonis area of hippocampus
CgC: cingulated cortex
CM: centromedial thalamic nucleus
DG: dentate gyrus of the hippocampus
dlST: dorsolateral striatum
DMH: dorsomedial hypothalamic nucleus
dmST: dorsomedial striatum
ENU: N-ethyl-N-nitrosourea
FITC: fluorescein isothiocyanate
Gad1: glutamate decarboxylase 1
GP: globus pallidus
LHb: lateral habenular nucleus
LS: lateral septum
MC: motor cortex
mPFC: the medial prefrontal cortex
Pir: piriform cortex
PH: posterior hypothalamus
PMCo: posteromedial cortical amygdaloid nucleus
PRh-Ent: perirhinal-entorhinal cortex
PT: paratenial thalamic nucleus
PTZ: pentylenetetrazole
SC: sensory cortex
SV2A: Synaptic vesicle glycoprotein 2A
Syt1: synaptotagmin1
TRITC: tetramethyrhodamine-5-(and 6)- isothiocyanate
VM: ventromedial thalamic nucleus

## References

[B1] Avoli M. de Curtis M. (2011). GABAergic synchronization in the limbic system and its role in the generation of epileptiform activity. *Prog. Neurobiol.* 95 104–132. 10.1016/j.pneurobio.2011.07.003 21802488 PMC4878907

[B2] Bastlund J. F. Berry D. Watson W. P. (2005). Pharmacological and histological characterization of nicotine-kindled seizures in mice. *Neuropharmacology* 48 975–983. 10.1016/j.neuropharm.2005.01.015 15857624

[B3] Chang W. P. Südhof T. C. (2009). SV2 renders primed synaptic vesicles competent for Ca2+-induced exocytosis. *J. Neurosci.* 29 883–897. 10.1523/JNEUROSCI.4521-08.2009 19176798 PMC2693337

[B4] Correa-Basurto J. Cuevas-Hernández R. I. Phillips-Farfán B. V. Martínez-Archundia M. Romo-Mancillas A. Ramírez-Salinas G. L. (2015). Identification of the antiepileptic racetam binding site in the synaptic vesicle protein 2A by molecular dynamics and docking simulations. *Front. Cell. Neurosci.* 9:125 10.3389/fncel.2015.00125 PMC4392693 25914622

[B5] Crèvecoeur J. Kaminski R. M. Rogister B. Foerch P. Vandenplas C. Neveux M. (2014). Expression pattern of synaptic vesicle protein 2 (SV2) isoforms in patients with temporal lobe epilepsy and hippocampal sclerosis. *Neuropathol. Appl. Neurobiol.* 40 191–204. 10.1111/nan.12054 23617838

[B6] Crowder K. M. Gunther J. M. Jones T. A. Hale B. D. Zhang H. Z. Peterson M. R. (1999). Abnormal neurotransmission in mice lacking synaptic vesicle protein 2A (SV2A). *Proc. Natl. Acad. Sci. U.S.A.* 96 15268–15273. 10.1073/pnas.96.26.15268 10611374 PMC24809

[B7] Custer K. L. Austin N. S. Sullivan J. M. Bajjalieh S. M. (2006). Synaptic vesicle protein 2 enhances release probability at quiescent synapses. *J. Neurosci.* 26 1303–1313. 10.1523/JNEUROSCI.2699-05.2006 16436618 PMC6674579

[B8] Feng G. Xiao F. Lu Y. Huang Z. Yuan J. Xiao Z. (2009). Down-regulation synaptic vesicle protein 2A in the anterior temporal neocortex of patients with intractable epilepsy. *J. Mol. Neurosci.* 39 354–359. 10.1007/s12031-009-92882 19757204

[B9] Franke H. Kittner H. (2001). Morphological alterations of neurons and astrocytes and changes in emotional behavior in pentylenetetrazol-kindled rats. *Pharmacol. Biochem. Behav.* 70 291–303. 10.1016/S0091-3057(01)006128 11701200

[B10] Fumoto N. Mashimo T. Masui A. Ishida S. Mizuguchi Y. Minamimoto S. (2014). Evaluation of seizure foci and genes in the Lgi1L385R/+ mutant rat. *Neurosci. Res.* 80 69–75. 10.1016/j.neures.2013.12.008 24406746

[B11] Herrera D. G. Robertson H. A. (1996). Activation of c-fos in the brain. *Prog. Neurobiol.* 50 83–107. 10.1016/S0301-0082(96)000214 8971979

[B12] Humpel C. Wetmore C. Olson L. (1993). Regulation of brain-derived neurotrophic factor messenger RNA and protein at the cellular level in pentylenetetrazol-induced epileptic seizures. *Neuroscience* 53 909–918. 10.1016/0306-4522(93)90476-V 8506025

[B13] Iha H. A. Kunisawa N. Tokudome K. Mukai T. Kinboshi M. Shimizu S. (2016). “Immunohistochemical analysis of Fos protein expression for exploring brain regions (foci) related to central nervous system (CNS) disorders and drug actions,” in *In Vivo Neuropharmacology and Neurophysiology*, ed. Philippu A. (New York, NY: Springer).

[B14] Janz R. Goda Y. Geppert M. Missler M. Südhof T. C. (1999). SV2A and SV2B function as redundant Ca2+ regulators in neurotransmitter release. *Neuron* 24 1003–1016. 10.1016/S0896-6273(00)810466 10624962

[B15] Kaminski R. M. Gillard M. Leclercq K. Hanon E. Lorent G. Dassesse D. (2009). Proepileptic phenotype of SV2A-deficient mice is associated with reduced anticonvulsant efficacy of levetiracetam. *Epilepsia* 50 1729–1740. 10.1111/j.1528-1167.2009.02089.x 19486357

[B16] Klitgaard H. Matagne A. Nicolas J. M. Gillard M. Lamberty Y. De Ryck M. (2016). Brivaracetam: rationale for discovery and preclinical profile of a selective SV2A ligand for epilepsy treatment. *Epilepsia* 57 538–548. 10.1111/epi.13340 26920914

[B17] Kudryashov I. E. Pavlova T. V. Kudryashova I. V. Egorova L. K. Gulyaeva N. V. (2007). Kindling in the early postnatal period: effects on the dynamics of age-related changes in electrophysiological characteristics of hippocampal neurons. *Neurosci. Behav. Physiol.* 37 765–772. 10.1007/s11055-007-0080-x 17922240

[B18] Löscher W. Ebert U. (1996). The role of the piriform cortex in kindling. *Prog. Neurobiol.* 50 427–481. 10.1016/S0301-0082(96)000366 9015822

[B19] Lynch B. A. Lambeng N. Nocka K. Kensel-Hammes P. Bajjalieh S. M. Matagne A. (2004). The synaptic vesicle protein SV2A is the binding site for the antiepileptic drug levetiracetam. *Proc. Natl. Acad. Sci. U.S.A.* 101 9861–9866. 10.1073/pnas.0308208101 15210974 PMC470764

[B20] Mashimo T. Yanagihara K. Tokuda S. Voigt B. Takizawa A. Nakajima R. (2008). An ENU-induced mutant archive for gene targeting in rats. *Nat. Genet.* 40 514–515. 10.1038/ng0508-514 18443587

[B21] Matveeva E. A. Vanaman T. C. Whiteheart S. W. Slevin J. T. (2007). Asymmetric accumulation of hippocampal 7S SNARE complexes occurs regardless of kindling paradigm. *Epilepsy Res.* 73 266–274. 10.1016/j.eplepsyres.2006.11.003 17174072 PMC1868484

[B22] Morgan J. I. Curran T. (1989). Stimulus-transcription coupling in neurons: role of cellular immediate-early genes. *Trends Neurosci.* 12 459–462. 10.1016/0166-2236(89)90096-9 2479148

[B23] Morimoto K. Fahnestock M. Racine R. J. (2004). Kindling and status epilepticus models of epilepsy: rewiring the brain. *Prog. Neurobiol.* 73 1–60. 10.1016/j.pneurobio.2004.03.009 15193778

[B24] Mukai T. Nagao Y. Nishioka S. Hayashi T. Shimizu S. Ono A. (2013). Preferential suppression of limbic Fos expression by intermittent hypoxia in obese diabetic mice. *Neurosci. Res.* 77 202–207. 10.1016/j.neures.2013.09.013 24144732

[B25] Nowack A. Yao J. Custer K. L. Bajjalieh S. M. (2010). SV2 regulates neurotransmitter release via multiple mechanisms. *Am. J. Physiol. Cell Physiol.* 299 C960–C967. 10.1152/ajpcell.00259.2010 20702688 PMC2980308

[B26] Ohno Y. Ishihara S. Mashimo T. Sofue N. Imaoku T. Shimizu S. (2011). Scn1a missense mutation causes limbic hyperexcitability and vulnerability to experimental febrile seizures. *Neurobiol. Dis.* 41 261–269. 10.1016/j.nbd.2010.09.013 20875856

[B27] Ohno Y. Ishihara S. Terada R. Kikuta M. Sofue N. Kawai Y. (2009a). Preferential increase in the hippocampal synaptic vesicle protein 2A (SV2A) by pentylenetetrazole kindling. *Biochem. Biophys. Res. Commun.* 390 415–420. 10.1016/j.bbrc.2009.09.035 19751703

[B28] Ohno Y. Okano M. Masui A. Imaki J. Egawa M. Yoshihara C. (2012a). Region-specific elevation of D1 receptor-mediated neurotransmission in the nucleus accumbens of SHR, a rat model of attention deficit/hyperactivity disorder. *Neuropharmacology* 63 547–554. 10.1016/j.neuropharm.2012.04.031 22580374

[B29] Ohno Y. Okumura T. Terada R. Ishihara S. Serikawa T. Sasa M. (2012b). Kindling-associated SV2A expression in hilar GABAergic interneurons of the mouse dentate gyrus. *Neurosci. Lett.* 510 93–98. 10.1016/j.neulet.2012.01.009 22266237

[B30] Ohno Y. Shimizu S. Harada Y. Morishita M. Ishihara S. Kumafuji K. (2009b). Regional expression of fos-like immunoreactivity following seizures in Noda epileptic rat (NER). *Epilepsy Res.* 87 70–76. 10.1016/j.eplepsyres.2009.07.012 19713079

[B31] Ohno Y. Shimizu S. Tatara A. Imaoku T. Ishii T. Sasa M. (2015). Hcn1 is a tremorgenic genetic component in a rat model of essential tremor. *PLoS ONE* 10:e123529 10.1371/journal.pone.0123529 PMC4430019 25970616

[B32] Paxinos G. Watson C. (2007). *The Rat Brain in Stereotaxic Coordinates*, 6th Edn Manhattan, NY: Elsevier.

[B33] Pollard J. R. (2008). Seletracetam, a small molecule SV2A modulator for the treatment of epilepsy. *Curr. Opin. Investig. Drugs* 9 101–107. 18183537

[B34] Racine R. J. (1972). Modification of seizure activity by electrical stimulation. II. Motor seizure. *Electroencephalogr. Clin. Neurophysiol.* 32 281–294. 10.1016/0013-4694(72)90177-0 4110397

[B35] Serajee F. J. Huq A. M. (2015). Homozygous mutation in synaptic vesicle glycoprotein 2A gene results in intractable epilepsy, involuntary movements, microcephaly, and developmental and growth retardation. *Pediatr. Neurol.* 52 642–646. 10.1016/j.pediatrneurol.2015.02.011 26002053

[B36] Szyndler J. Maciejak P. Turzyńska D. Sobolewska A. Taracha E. Skórzewska A. (2009). Mapping of c-Fos expression in the rat brain during the evolution of pentylenetetrazol-kindled seizures. *Epilepsy Behav.* 16 216–224. 10.1016/j.yebeh.2009.07.030 19713157

[B37] Toering S. T. Boer K. de Groot M. Troost D. Heimans J. J. Spliet W. G. (2009). Expression patterns of synaptic vesicle protein 2A in focal cortical dysplasia and TSC-cortical tubers. *Epilepsia* 50 1409–1418. 10.1111/j.1528-1167.2008.01955.x 19220410

[B38] Tokudome K. Okumura T. Shimizu S. Mashimo T. Takizawa A. Serikawa T. (2016). Synaptic vesicle glycoprotein 2A (SV2A) regulates kindling epileptogenesis via GABAergic neurotransmission. *Sci. Rep.* 6:27420 10.1038/srep27420 PMC4893657 27265781

[B39] van Vliet E. A. Aronica E. Redeker S. Boer K. Gorter J. A. (2009). Decreased expression of synaptic vesicle protein 2A, the binding site for levetiracetam, during epileptogenesis and chronic epilepsy. *Epilepsia* 50 422–433. 10.1111/j.1528-1167.2008.01727.x 18717715

[B40] Xu T. Bajjalieh S. M. (2001). SV2 modulates the size of the readily releasable pool of secretory vesicles. *Nat. Cell Biol.* 3 691–698. 10.1038/35087000 11483953

